# ARE OLDER ADULTS’ PERCEPTIONS OF ELDERSPEAK INFLUENCED BY SELF-REPORTED PSYCHOSOCIAL CHARACTERISTICS?

**DOI:** 10.1093/geroni/igad104.3180

**Published:** 2023-12-21

**Authors:** Lisa Weimar, Clarissa Shaw

**Affiliations:** University of Iowa, Iowa City, Iowa, United States; University of Iowa, Iowa City, Iowa, United States

## Abstract

PROMIS (Patient-Reported Outcomes Measurement Information System) is a system of highly reliable measures of patient-reported health status. Elderspeak is speech that sounds childish to older adults. Use of elderspeak by nursing staff can result in rejection of care. The Iowa Coding Scheme for Elderspeak (ICodE) contains 11 elderspeak attributes: diminutives, childish terms, collectives, short words, directives, reflectives, minimizers, exaggerated praise, laughter, tag questions and exaggerated prosody. Thirty-one older adults (aged 74.5±7.8 years) responded to short form PROMIS instruments measuring self-efficacy, physical function, isolation, cognitive function, depression, anxiety, and pain interference. Participants listened to 41 audio recordings of nursing staff providing care to hospitalized patients with dementia and were asked to rate each audio recording using a 5-point Emotional Tone Rating Scale for patronizing and respectful (5=very, 1=not at all). Using the ICodE, each audio recording was classified as neutral (no elderspeak), containing a single attribute, or containing an attribute combined with exaggerated prosody. Ratings for elderspeak attributes ranged from 2.1 to 2.9 for patronizing and 2.8 to 3.8 for respectful. Using the SPSS statistical package, a Spearman’s rho correlation was calculated for each elderspeak rating and health status domain identified above. Among this small, homogenous sample, no statistically significant correlation was found between participants’ self-efficacy, isolation, physical or cognitive function, depression, anxiety, or pain interference, as measured by PROMIS tools, and their rating of elderspeak attributes as respectful or patronizing. Future research should continue to explore what factors influence older adults’ perceptions of elderspeak as being respectful or patronizing.

